# Evaluating the Processing and Nutrient Digestibility of Defatted *Nannochloropsis* sp. QH25 Co-Product for Sustainable Rainbow Trout Aquaculture Diets

**DOI:** 10.3390/microorganisms14092104

**Published:** 2026-09-20

**Authors:** Pallab K. Sarker, Benjamin V. Schoffstall, Anne R. Kapuscinski, Devin Fitzgerald, Anindita Datta, Duncan Gwynne, Connor Greenwood

**Affiliations:** 1 Environmental Studies Department, University of California Santa Cruz, Santa Cruz, CA 95064, USA; bschoffs@ucsc.edu (B.V.S.); akapusci@ucsc.edu (A.R.K.); adatta10@ucsc.edu (A.D.); dgwynne@ucsc.edu (D.G.); 2 California Sea Grant Extension Program, University of California San Diego, San Diego, CA 92093, USA; defitzgerald@ucsd.edu; 3 Monterey Bay Salmon and Trout Project, Big Creek Rd., Davenport, CA 95017, USA; cdgreenw@ucsc.edu

**Keywords:** aquafeed, microalgae, *Nannochloropsis* sp. QH25, defatted co-product, nutrient digestibility, extrusion, rainbow trout

## Abstract

The sustainability limitations of fishmeal and fish oil have increased interest in nutrient- rich microalgal co-products for aquafeeds. This study evaluated the effects of processing on the nutritional composition and apparent digestibility of a defatted *Nannochloropsis* sp. QH25 co-product in rainbow trout (*Oncorhynchus mykiss*). Experiment 1 assessed the effects of extrusion at 90 °C or 127 °C, with or without precooking, and enzymatic treatment with protease, xylanase, and β-glucanase. Based on nutrient composition, the 90 °C extrusion treatment without precooking was selected for Experiment 2, which compared a reference diet with three diets containing 30% raw, extruded, or enzyme-treated co-product, using four replicate tanks per diet. Extrusion at 127 °C reduced crude protein, gross energy, and methionine concentrations, whereas extrusion at 90 °C largely preserved nutrient composition. Precooking reduced several essential amino acids but increased methionine and did not affect tryptophan. Crude protein digestibility was numerically higher in the extruded co-product than in the raw co-product, although the difference was not statistically significant. Most essential amino acids showed similar digestibility in the raw and extruded treatments, whereas enzymatic processing resulted in lower crude protein and amino acid digestibility. Omega-3 fatty acid digestibility remained high across diets. Overall, the raw co-product showed high nutrient digestibility, while extrusion resulted in only small, generally non-significant numerical differences and enzymatic processing generally resulted in lower nutrient digestibility under the conditions evaluated.

## 1. Introduction

Aquaculture has expanded and has the potential to provide affordable and nutritious high-protein food to meet the needs of the world’s growing population. The global aquaculture production has reached 112 Mt in 2017 [1]. However, for continuous expansion, there is a need for cost-effective alternatives to traditional marine feedstuffs such as fishmeal and fish oil. These have been widely used in aquafeeds due to their nutritional values [1,2]. Pelagic fish like anchovy, herring, and menhaden have fishmeals with high protein levels and an ideal balance of amino acids. Fish oils also contain high concentrations of long-chain polyunsaturated fatty acids (LC-PUFAs) of the n-3 series. Unfortunately, the supply of fishmeal and fish oil derived from these marine pelagic fishes has remained stagnant, while demand, particularly from the aquaculture sector, has increased [3]. As a result, the prices for fishmeal and fish oil have more than doubled since the 2000s and have continued to rise steadily over the past decade [1]. Each year, approximately 16 million tons of wild forage fish, much of which is suitable for direct human consumption, are used to produce fishmeal (FM) and fish oil (FO). Aquaculture accounts for approximately 87% of global fishmeal use and 74% of global fish oil use [4,5]. This reliance is particularly relevant given that global compound aquafeed use was projected to increase from 51.23 million tonnes in 2017 to 73.15 million tonnes by 2025 [6]. Therefore, reducing the use of fishmeal and fish oil in aquafeeds and developing alternative feed ingredients could help meet the feed demands of aquaculture [1]. To reduce the over-extraction of wild fish resources, aquafeed often contains plant-based ingredients which can be nutritionally deficient due to low digestibility, high levels of anti-nutritional factors, and a lack of essential amino acids like lysine, methionine, threonine, and tryptophan as pointed out by [7]. However, it is particularly challenging to reduce the reliance on fishmeal in the diets of salmonids as these carnivorous fish require high levels of protein, amino acids, and fatty acids for their growth and health, as highlighted by [8,9].

Microalgae have emerged as one of the most promising sustainable sources of alternative protein and lipid ingredients for aquafeeds [10,11,12,13,14,15]. Compared with many conventional alternative feed ingredients, microalgae offer several advantages, including high concentrations of protein, long-chain polyunsaturated fatty acids (LC-PUFAs), or both, while being amenable to large-scale production under controlled cultivation systems [14,16,17,18]. Unlike fishmeal and fish oil, microalgal biomass can be produced independently of wild fisheries, providing a more environmentally sustainable and scalable source of aquafeed ingredients.

Commercial production of microalgae for biofuels, nutraceuticals, and high-value lipids also generates substantial quantities of defatted co-products following oil extraction. Although these residual biomasses are often treated as low-value waste streams, they retain considerable nutritional value, particularly as protein-rich feed ingredients suitable for aquaculture [18]. Incorporating microalgal co-products into aquafeeds may promote more complete utilization of microalgal biomass [14,16]. In Atlantic salmon, dietary inclusion of DHA-rich *Schizochytrium* sp. improved EPA and DHA retention efficiency and certain measures of fillet quality without adversely affecting growth performance [19]. Microalgae can also contain valuable compounds, including pigments, β-glucans with potential immunostimulatory properties, and other bioactive metabolites [20]. The recovery and marketing of multiple biomass fractions may improve the economic viability of microalgal biorefineries [14,16].

Recent findings further underscore the potential of *Nannochloropsis* co-products, demonstrating that this biomass can fully replace fishmeal in rainbow trout diets while maintaining growth performance and providing potential environmental benefits [18]. In a separate study, replacing fishmeal with this co-product reduced biotic-resource use, although these benefits were accompanied by trade-offs, including increased land and water use [21].

Despite this potential, the rigid cell wall and complex non-starch polysaccharides of *Nannochloropsis* may restrict nutrient accessibility and reduce nutrient and energy digestibility, providing a rationale for evaluating processing strategies [22]. Extrusion conditions have been shown to influence the nutritional composition and digestibility of defatted *Nannochloropsis oculata* co-product in rainbow trout [22]. Andrade et al. [23] reported that phosphorus digestibility did not differ significantly between the enzyme-treated and reference diets. Among the diets tested, the enzyme-treated diet had the highest mean phosphorus retention and the lowest mean dissolved phosphorus excretion; however, the authors noted that these outcomes may partly reflect its lower phosphorus intake [23]. More broadly, extrusion conditions can affect the physical and nutritional characteristics of aquafeeds and the digestibility and utilization of feed ingredients [24].

Despite these advances, it remains unclear whether extrusion or enzymatic processing is necessary to improve the nutritional value and nutrient digestibility of defatted *Nannochloropsis* sp. QH25 co-product.

In this study, we developed novel protein ingredients from defatted *Nannochloropsis* sp. QH25 co-product (the residual biomass remaining after oil extraction) using extrusion and enzymatic processing to enhance its nutritional value and suitability for aquafeed applications. Based on the processing evaluation, the low-temperature extrusion-treated co-product was selected for digestibility assessment. The apparent digestibility of nutrients in the raw and extrusion-processed co-products was then determined in rainbow trout (*Oncorhynchus mykiss*). This study provides a comprehensive evaluation of processing strategies to determine whether processing enhances nutrient digestibility and highlights the potential of valorizing defatted *Nannochloropsis* co-products as sustainable alternatives to conventional aquafeed ingredients.

## 2. Materials and Methods

### 2.1. Overall Experimental Design

**Figure 1:**
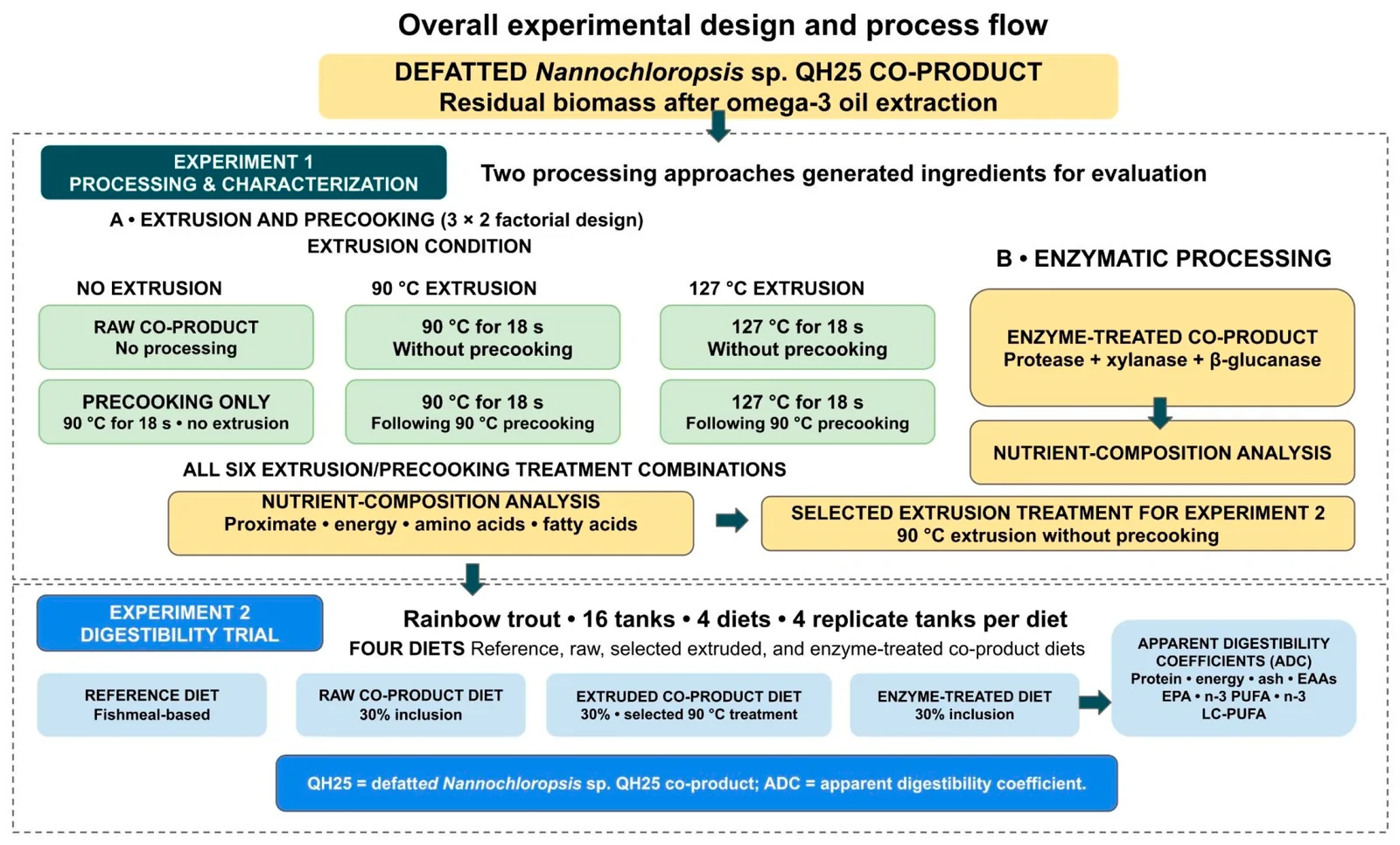
Overall experimental design and process flow. Experiment 1 produced and characterized raw, extruded, and enzyme-treated *Nannochloropsis* sp. QH25 co-products. The selected extrusion treatment (90 °C), along with the raw and enzyme-treated co-products, were subsequently evaluated and compared with a reference diet in a rainbow trout digestibility trial in Experiment 2.

The study comprised two linked experiments (Figure 1). Experiment 1 evaluated the processing and nutritional composition of defatted *Nannochloropsis* sp. QH25 co-product using two processing approaches: extrusion combined with precooking and enzymatic treatment. The extrusion and precooking component followed a 3 × 2 factorial design comprising three extrusion conditions—no extrusion, extrusion at 90 °C and extrusion at 127 °C—and two precooking conditions: without precooking and with precooking at 90 °C for 18 s. This design produced six treatment combinations: raw co-product, precooking alone, 90 °C extrusion without precooking, 90 °C extrusion following precooking, 127 °C extrusion without precooking and 127 °C extrusion following precooking. A separate portion of the raw co-product was treated enzymatically with protease, xylanase and β-glucanase. Nutrient composition was analyzed in all six extrusion/precooking treatment combinations and in the enzyme-treated co-product. Based on the compositional results, extrusion at 90 °C without precooking was selected as the extrusion treatment for Experiment 2.

Experiment 2 evaluated nutrient digestibility in rainbow trout using four diets: a fishmeal-based reference diet and three test diets containing 30% raw, selected extruded or enzyme-treated QH25 co-product. The digestibility trial comprised 16 tanks, with four replicate tanks assigned to each diet. Apparent digestibility coefficients were determined for protein, energy, ash, essential amino acids, EPA, total n-3 PUFA and total n-3 LC-PUFA.

### 2.2. Processing Nannochloropsis sp. QH25 Co-Product

#### 2.2.1. Extrusion Processing Experiment for *Nannochloropsis* sp. QH25 Co-Product

The six extrusion and precooking treatment combinations described in Section 2.1 and Figure 1 were produced following the methods described by Sarker et al. [22]. Four treatments underwent extrusion at either 90 °C or 127 °C, with or without precooking at 90 °C, while the raw and precooking-only treatments served as non-extruded comparisons.

The *Nannochloropsis* sp. QH25 co-product was processed at the Kapuscinski-Sarker Lab located in Natural Science II (University of California, Santa Cruz, CA, USA) using a single-screw extruder (TT-100 tabletop lab scale extruder from Akron Tool and Die, Akron, OH, USA). The co-product used in the extrusion without precooking group was exposed to an average temperature of 90 °C or 127 °C through the extruder barrel for 18 s. In the precooking treatments, the co-product was precooked as a dry mash at 90 °C and passed through the same single-screw extruder for 18 s. After extrusion the co-product was ground for use. The pressure at the die head varied in all the treatments but averaged 1.04 ± 0.18 PSI depending on the screw speed and diet moisture. Depending on the consistency of the mash we adjusted the screw speed to ensure the 18.0 ± 0.17 s barrel retention time was met. The average motor RPM speed was 35.5–40.8% for high-moisture mash (>30%) and 40.8–50% for lower-moisture mash (<25%). After it was ground we collected samples from each treatment for chemical analysis. Qualitas Health, Inc. donated the defatted *Nannochloropsis* sp. QH25 co-product, consisting of the residual biomass remaining after oil extraction.

#### 2.2.2. Enzymatic Processing of *Nannochloropsis* sp. QH25 Co-Product

We selected three enzymes—protease, xylanase, and β-glucanase—to evaluate their potential effects on nutrient composition and digestibility, based on findings from previous studies [22].

Dalsgaard et al. [25] previously evaluated β-glucanase, xylanase, protease, and their combination in an extruded soybean-based diet for rainbow trout. The enzymes were thoroughly mixed with the dry co-product alone until homogenous at the following dosages (mg enzyme/kg co-product): protease (serine protease enzyme; DSM Nutritional Products, Wilhelminasingel, MA, USA) at 228 mg/kg; xylanase (RONOZYME1 WX L, DSM Nutritional Products, Wilhelminasingel, MA, USA) at 208 mg/kg; and glucanase (β-glucanase, RONOZYME1 VP (L), DSM Nutritional Products, Wilhelminasingel, MA, USA) at 67 mg/kg [22]. This combination of enzymes and dosage levels has been determined from previous experimental results [22,26]. No preliminary optimization trials or separate enzymatic incubation under controlled pH, temperature, and time conditions were conducted prior to extrusion in the present study. Following diet formulation, all three test diets were extruded and steam-pelleted at 90 °C; therefore, the enzyme-treated diet was exposed to both heat and pressure. The co-product used in the extruded diet had already been extruded separately at 90 °C before diet formulation and was consequently exposed to processing twice.

### 2.3. Digestibility Experiment, Fish Husbandry, and Fecal Collection

#### 2.3.1. Dietary Design

This digestibility experiment compared a reference diet to three experimental diets with raw, extruded, or enzyme-processed *Nannochloropsis* sp. QH25 co-product. Based on the extrusion processing results, the 90 °C no precooking treatment was selected for the extruded diet. Overall nutrient content in the 90 °C no precooking treatment did not differ from the raw treatment, but cooking and high-temperature extrusion led to several nutrient deficiencies (described in Section 3). Thus, based on the nutritional composition observed in Experiment 1 and our previous findings [22], the 90 °C extrusion treatment without precooking was selected for the subsequent digestibility experiment. Extrusion at 127 °C reduced crude protein, gross energy, and methionine, while precooking reduced fat, gross energy, and several essential amino acids (included in Section 3). In addition, our previous study showed lower methionine concentration following extrusion at 127 °C compared with 90 °C. Therefore, the 90 °C extrusion condition was selected to minimize potential heat-related nutrient losses while providing a practically relevant processing condition for the digestibility evaluation. Our primary focus for these diets is to evaluate the apparent digestibility coefficient (ADC) of protein, lipid, energy, essential amino acids, and fatty acids in the microalgal co-product ingredients and test diets (Table 1). The test diets were formulated using a 70:30 ratio of reference diet to test ingredient (as-is basis), following established protocols for determining the apparent digestibility of novel feed ingredients in fish [13,14,16,22,27,28,29]. The 30% inclusion level was selected to provide a sufficient contribution of the test ingredient for reliable estimation of its digestible nutrient contribution while maintaining the nutritional characteristics of the reference-based test diet. This inclusion level was used specifically for the digestibility assay and should not be interpreted as a recommended commercial inclusion level of the QH25 co-product in practical aquafeeds. We included an indigestible marker, yttrium oxide, from Thermo Scientific, Waltham, MA, USA, in the basal diet at 1% as a digestion indicator [22]. We thoroughly mixed the microingredients before combining them with the macroingredients to create a fully homogenized mixture. The extruder at the Kapuscinski-Sarker Lab was utilized to steam pellet the mixture. During extrusion, the diet was exposed to an average target temperature in the barrels at 90 °C and passed through the extruder for 18 s exposure. All diets were top-coated with fish oil using a rotating mixer (SUNCOO 4/5HP Electric Concrete Cement Mixer, 5 Cu Ft Mortar Mixing Stucco Seeds Portable Barrow Machine) for approximately 15 min to ensure even coating. After mixing, the feed was dried overnight in a fume hood to reduce the moisture content to approximately 16–18%. The pellets were sieved to remove excess dust and stored at –20 °C.

#### 2.3.2. Fish Husbandry

Rainbow trout were randomly distributed across the 16 freshwater recirculating aquaculture systems (RASs) located in the aquaculture greenhouse at the University of California, Santa Cruz, CA, USA. Each 757 L tank started with 12–14 juvenile rainbow trout with an average weight of 46.3 ± 1.4 g, sourced from Thomas Fish Company (Anderson, CA, USA). The fish were fed the reference diet for 7 days to acclimate to the systems before being randomly assigned treatments. The digestibility trial and fecal collection were conducted over a 14-day period.

Four dietary treatments were evaluated: a reference diet, a diet containing the raw ingredient, a diet containing the enzyme-treated ingredient, and a diet containing the extruded ingredient. Each diet was allocated to four replicate tanks (*n* = 4), resulting in 16 experimental tanks, and fed according to restricted pair feeding to supply sufficient nutrients to all groups [13,16,22,28]. Twelve to 14 fish were maintained in each tank, with an average of approximately 13 fish per tank. Fish were randomly allocated among the experimental tanks before the feeding trial. The number of fish per tank was maintained within the 12–14 range across treatments, and all other husbandry and water-quality conditions were maintained consistently among experimental tanks. Thus, dietary treatment was the primary experimental factor intentionally varied among groups. Tanks were fed to apparent satiation twice a day between 0930 and 1600 h. Throughout the trial we measured the dissolved oxygen (mg/L), dissolved oxygen saturation (%), temperature, and pH daily utilizing the YSI Pro1020 handheld multiparameter meter. Total ammonia nitrogen, nitrite nitrogen, and nitrate nitrogen were tested weekly using a YSI 9500 photometer to ensure favorable conditions for rainbow trout. Average conditions during the trial were as follows: water temperature 15.4 °C, pH 8.6, dissolved oxygen 8.7 mg/L, total ammonia nitrogen 0.2 mg/L, nitrite nitrogen 0.1 mg/L, and nitrate nitrogen 26.8 mg/L.

#### 2.3.3. Fecal Collection

Feces were collected from each tank daily prior to feed using an approach based on the Guelph system to minimize nutrient leaching [27]. A radial flow settler installed on the side of each RAS that acted as a modified settlement column allowed us to collect intact fecal material from the bottom of the settler [22]. The settled solids were removed via siphoning into a dedicated collection bin, where intact fecal pellets were carefully removed using pipettes into a 50 mL Falcon Tube (Corning, Corning, NY, USA); [9,14]).

### 2.4. Chemical Analysis and Calculations

Previous research from our lab guided the digestibility calculations and chemical analysis procedures [9,14,22,23]. We analyzed *Nannochloropsis* sp. QH25 co-product, test diets, and feces for proximate, amino acid, and fatty acid content (Table 2 and Table 3). These samples were analyzed at New Jersey Feed Laboratory, Inc. (Ewing, NJ, USA) according to the following methods: moisture (AOAC 930.15), crude protein (AOAC 990.03), crude lipid (AOAC 920.39), ash (AOAC 942.05), crude fiber (AOAC 978.10), and gross energy (determined using an automated oxygen bomb calorimeter). Amino acid profiles were quantified using high-performance liquid chromatography (HPLC) with an Agilent 1260 Infinity according to AOAC methods 994.12, 985.28, 988.15, and 994.12. Fatty acid composition was determined as fatty acid methyl esters (FAME) following AOAC method 963.22.

The ADCs of proximate components, amino acids, and fatty acids in the microalgal ingredients and test diets were calculated using the methods described in [26,30,31]:

$${ADC}_{diet}\;=\;[1\;-\;(\frac{F}{D})\;\times \;(\frac{D_{i}}{F_{i}})]$$

$${ADC}_{test\;ingredient}={ADC}_{test\;diet}+({ADC}_{test\;diet}-{ADC}_{ref\;diet})\times \frac{0.7D_{ref}}{0.3D_{ingredient}}$$

where:

*D* = % nutrient (or kJ g 1 gross energy) of diet;

*F* = % nutrient (or kJ g 1 gross energy) of feces;

*D_i_* = % digestion indicator (yttrium oxide) of diet;

*F_i_* = % digestion indicator (yttrium oxide) of feces.

### 2.5. Statistics

For the extrusion processing experiment we used a mean ± standard error of macronutrients and essential amino acids in co-product form from the 2 by 3 factorial treatment design. For the digestibility experiment, apparent digestibility coefficients (ADCs) of macronutrients, essential amino acids, and fatty acids in the reference and test diets were analyzed using a one-way analysis of variance (ANOVA), with dietary treatment as the fixed factor. The experimental tank was considered the experimental unit, as fish within the same tank shared the same dietary treatment and rearing environment and therefore were not considered independent observations. Accordingly, the four replicate tanks per dietary treatment constituted the independent replicates (*n* = 4), and data were analyzed at the tank level. All data were tested for ANOVA assumptions prior to proceeding with analysis. Specifically, normality was assessed using the Shapiro–Wilk test, and homogeneity of variances was assessed using Levene’s test. When a significant treatment effect was detected (*p* < 0.05), treatment means were separated using Tukey’s multiple-comparison test at a 95% confidence level. Results are presented as mean ± standard error (SE) of the four replicate tanks. All statistical analyses were performed using IBM SPSS Statistics (v. 31.0, IBM Corp., Armonk, NY, USA). This statistical approach is consistent with previously published digestibility studies in tilapia and rainbow trout [14,16,22,23,26].

## 3. Results

### 3.1. Extrusion Processing

Most of the proximate composition values, except for fiber (*p* = 0.07), were numerically higher (*p* < 0.05) in the non-extruded co-product and significantly lower in the high thermal extrusion co-product (Table 4). Compared to non-cooking, cooking the algal co-product also significantly affected most of the proximate composition values, except for dry matter (*p* = 0.09) and protein (*p* = 0.13). Interaction effects between extrusion and cooking were only detected in the fat content (*p* < 0.001).

In the extrusion group, the non-extruded treatment proximate composition parameter values were significantly higher in dry matter (91.8–93.9%, *p* < 0.001), protein (44.7–45.9%), carbohydrates (14.5–15.1%, *p* < 0.001), ash (31.1–31.8%, *p* < 0.001) and energy (2148.6–2188.5 kcal, *p* < 0.001) compared to the low- and high-extruded treatments. The fat content was significantly higher (1.2–1.3%, *p* = 0.07) in the high-thermal-extruded treatment. The fiber content did not differ significantly (0.8–1.3%, *p* = 0.07) across treatments but was numerically highest in the non-extruded treatment (Table 4).

In the precooking group, the dry matter (92.7–93.0%, *p* = 0.09) and protein (45.2–45.5%, *p* = 0.13) were higher in the non-cook treatment but did not significantly differ from the cooking treatment. The fat (1.1–1.3%, *p* < 0.001) and ash (31.3–31.6%, *p* = 0.001) contents were significantly higher in the non-cooked treatment than in the cooking treatment. The carbohydrate (14.6–14.9%, *p* < 0.001) and energy (2154.8–2185.0 kcal, *p* < 0.001) contents were significantly higher in the cooking treatment compared to the non-cooked treatment (Table 4).

The amino acid content of the co-product was not significantly impacted by extrusion, except that methionine (*p* < 0.001) was significantly higher in the non-extruded treatment (Table 5). However, precooking significantly impacted (*p* < 0.05) most of the amino acids. Interaction effects between the extrusion and cooking groups were detected in most amino acids except for tryptophan (*p* = 0.38).

The thermally extruded treatments in the extrusion group had the same lysine (2.4–2.5%, *p* = 0.46) and threonine (2.1–2.2%, *p* = 0.21) levels but did not differ significantly from each other or the non-extruded treatment. The methionine content (0.74–0.90%, *p* < 0.001) in the non-extruded treatment was significantly higher than that of the thermally extruded groups.

Precooking led to a significant reduction (*p* < 0.05) in most amino acid levels, except for a significant increase in methionine (0.80–0.86%, *p* = 0.002) and an equal level of tryptophan (0.7–0.7%, *p* = 0.6). The lysine (2.4–2.5%, *p* = 0.005) and threonine (2.1–2.2%, *p* = 0.003) levels were significantly higher in the non-cooking treatment.

### 3.2. Digestibility of Proximate Composition, Fatty Acids, and Amino Acids in Test Nannochloropsis sp. QH25 Ingredients

We detected significantly higher digestibility of protein (84.6%, *p* < 0.05) in the extruded treatment compared to the enzyme-processed treatment (Table 6). The digestibility of protein in the raw *Nannochloropsis* sp. QH25 was not significantly different (*p* > 0.05) compared to the enzyme and extruded treatments. The digestibility of ash was significantly higher in the raw co-product (*p* < 0.05); carbohydrates and calories did not vary significantly but were all higher in the raw treatment (*p* > 0.05).

The digestibility of most amino acids in the enzyme treatment was significantly lower than that in the raw and extruded treatments. Methionine and lysine digestibility were highest in the raw ingredient (87.1% and 86.2%, respectively), whereas threonine digestibility was highest in the extruded ingredient (78.42%).

We analyzed the digestibility of the total n3 PUFA, eicosapentaenoic (EPA) and total n3 LCPUFA in the raw and processed ingredients (Table 6). The total n3 PUFA and EPA were highest in the raw microalgal co-product but did not significantly differ (*p* > 0.05) compared to the extruded treatment. The total n3 LCPUFA was not significantly different across treatments (*p* > 0.05), but was highest in the raw algal co-product compared to the extruded and enzyme-treated treatments.

### 3.3. Digestibility of Proximate Composition, Fatty Acids, and Amino Acids in Reference and Test Diets

We reported the digestibility of proximate analysis, amino acids, and fatty acids in the diets in Table 7. The protein was significantly higher (*p* < 0.01) in the reference diet (93.2%) compared to the experimental diets. Amongst the experimental diets, the protein level was significantly lower (*p* < 0.05) in the enzyme diet (88.5%) compared to the extrusion (90.32%) and raw (89.9%) diets.

The digestibility of most essential amino acids was significantly higher (*p* < 0.05) in the reference diet than the enzyme diet. A majority of essential amino acids were not significantly different between the reference, raw, and extruded treatments. However, the extruded treatment had the lowest cystine digestibility (79.3%) compared to the other experimental diets (*p* < 0.05). The methionine content was significantly higher (93.8%, *p* < 0.01) in the reference diet compared to the experimental diets. The methionine levels did not differ significantly (*p* > 0.05) between the extrusion and raw diets. However, the methionine content was significantly lower (*p* < 0.05) in the enzyme diet compared to the other diets. The lysine content was significantly lower (*p* < 0.01) in the enzyme diet compared to the other diets. The lysine content did not significantly differ (*p* > 0.05) in the reference, extrusion and raw diets. The threonine level was significantly lower (75.71%, *p* < 0.01) in the enzyme diet compared to the other diets. The threonine levels did not differ significantly (*p* > 0.05) between the reference, extrusion and raw diets.

The digestibility of the omega-3 fatty acid content in the test diets showed significant differences (*p* < 0.05). The total n3 PUFA content was significantly higher (98.4%, *p* < 0.01) in the raw diet compared to the enzyme and extruded diet, but did not differ significantly (*p* > 0.05) from the reference diet. The EPA content was significantly lower (96.5%, *p* < 0.01) in the enzyme diet compared to the reference (98.6%) and raw (98.8%) diets, but did not differ significantly (*p* > 0.05) compared to the extrusion diet (97.7%). Total n-3 LC-PUFA digestibility was significantly higher in the reference (98.0%) and raw (98.3%) diets than in the enzyme diet (95.9%). The extruded diet had an intermediate value (97.1%) that did not differ significantly from any of the other diets (*p* = 0.006).

## 4. Discussion

This study evaluated the effects of extrusion and enzymatic processing on the nutritional composition and digestibility of defatted *Nannochloropsis* sp. QH25 co-product for rainbow trout. Overall, the present study demonstrates that the QH25 co-product is a nutritionally valuable ingredient for rainbow trout feeds, exhibiting high digestibility of protein, essential amino acids, and omega-3 fatty acids. Although processing treatments influenced nutrient composition, particularly the retention of specific nutrients, extrusion at 90 °C did not substantially enhance or diminish the overall nutritional quality of the QH25 co-product. In contrast, enzymatic processing consistently reduced nutrient utilization under the conditions evaluated. Collectively, the nutrient composition and digestibility results indicate that raw and extrusion-treated QH25 co-products provide comparable nutritional value, supporting the potential of the raw co-product as a sustainable ingredient for aquafeed applications.

### 4.1. Effect of Extrusion Temperature and Precooking on the Nutritional Composition of Nannochloropsis sp. QH25 Co-Product

Extrusion at 90 °C largely preserved the nutritional quality of QH25 co-product, whereas extrusion at 127 °C reduced crude protein, gross energy, and methionine (Table 4 and Table 5). Similar responses were reported by Sarker et al. [22] who also observed lower methionine concentrations following extrusion at 127 °C than at 90 °C in *Nannochloropsis* co-product. Likewise, Singh et al. [31] demonstrated that increasing extrusion severity reduced the retention of sulfur-containing amino acids, with methionine being among the most heat-sensitive. Consistent with these studies, methionine was the only essential amino acid significantly affected by extrusion temperature, whereas the remaining essential amino acids were largely unchanged.

Although the fiber content decreased numerically with increasing extrusion temperature (Table 4), the observed trend is consistent with previous studies describing structural modification of *Nannochloropsis* during extrusion. The cell wall of *Nannochloropsis* contains a cellulose-rich inner layer and a chemically resistant, hydrophobic algaenan-containing outer layer; hemicellulose has also been identified among its structural components [32,33].

Wang et al. [34] reported that single-screw extrusion disrupted the cell walls of *Nannochloropsis oceanica* through heat, compression, mixing, and shear forces. They observed structural damage to the cells and increased recovery of intracellular lipids and sugars. Although cell-wall disruption was not evaluated directly in the present study, the numerical reduction in fiber and increased digestibility following extrusion could be consistent with partial cell-wall disruption.

Precooking exerted a greater influence on nutrient composition than extrusion temperature alone. Although crude protein remained unchanged, precooking reduced fat, gross energy, and several essential amino acids (Table 4 and Table 5). Similar observations have been reported in previous studies, where lysine was identified as the amino acid most susceptible to thermal processing because its reactive ε-amino group readily participates in Maillard reactions, while severe heating also reduced the retention of other essential amino acids [31,35]. Significant extrusion temperature × precooking interactions further indicate that amino acid retention depended on the combination of thermal treatments rather than either treatment alone. Because precooking reduced several nutritionally important amino acids without improving crude protein concentration, digestibility was evaluated only in the non-precooked co-products to isolate the effects of extrusion temperature.

### 4.2. Effect of Processing on Nutrient Digestibility

Processing affected nutrient digestibility differently depending on the processing method (Table 6 and Table 7). The principal finding of the present study was that there were no statistically significant differences between the raw and extrusion-treated (90 °C) *Nannochloropsis* sp. QH25 co-products for nearly all nutrient digestibility parameters. The study compared the raw defatted QH25 co-product with the same co-product after extrusion at 90 °C and after enzymatic processing. Although extrusion at 90 °C resulted in numerically greater apparent digestibility coefficients for crude protein and several essential amino acids, the magnitude of these improvements was modest and did not differ significantly from those observed for the raw co-product. In contrast, enzymatic processing consistently reduced nutrient utilization. These findings indicate that, under the processing conditions evaluated, moderate extrusion resulted in only small, non-significant increases in nutrient digestibility compared with the raw co-product. Thus, the raw QH25 co-product exhibited nutrient digestibility comparable to that of the moderately extruded co-product.

Extrusion has been widely used to improve nutrient availability from microalgal ingredients because heat, pressure, and mechanical shear disrupt the resistant cell wall and increase access to intracellular nutrients [34]. Tibbetts et al. [36] reported higher in vitro-predicted protein digestibility for *Nannochloropsis granulata* meals after supercritical-fluid extraction at 70 and 90 °C than for the untreated biomass. Similarly, Sarker et al. [22] reported a higher apparent digestibility of crude protein, energy, and several amino acids in extruded *Nannochloropsis oculata* co-product than in the raw co-product under the conditions evaluated. In contrast, the crude protein digestibility of the extruded *Nannochloropsis* sp. QH25 co-product did not differ significantly from that of the raw co-product. This suggests that nutrient accessibility in the raw QH25 co-product was already sufficiently high and that additional cell-wall disruption through moderate extrusion produced little measurable improvement [36]. Differences among studies likely reflect variation in *Nannochloropsis* species, co-product composition following oil extraction, and extrusion conditions, all of which influence the response of microalgal ingredients to thermal processing [24].

A similar pattern was observed for essential amino acids. At the ingredient level, the digestibility of most essential amino acids did not differ significantly between the raw and extruded co-products, whereas the enzyme-treated co-product generally exhibited lower digestibility. At the diet level, the digestibility of lysine, isoleucine, threonine, histidine, arginine, and tryptophan did not differ significantly among the raw, extruded, and fishmeal reference diets. These findings are consistent with Sørensen et al. [15], who reported that defatted *Nannochloropsis oceanica* meal provided digestible nutrients to Atlantic salmon. They also reinforce the importance of evaluating digestibility because nutrient composition alone does not establish the availability of nutrients from microalgal ingredients [36].

Previous studies have reported variable effects of extrusion on nutrient digestibility. Sarker et al. [22] found higher apparent digestibility of crude protein, energy, and several amino acids in extruded *Nannochloropsis oculata* co-product than in the raw co-product. Le Boucher et al. [37] found that a lower extrusion temperature increased the digestibility of several essential amino acids in barramundi diets, although protein, lipid, and energy digestibility were not significantly affected by the extrusion settings. Together with the present results, these findings indicate that the effects of extrusion on amino acid digestibility can vary with ingredient characteristics and processing conditions [24,37].

Omega-3 fatty acids exhibited a slightly different response to processing. At the diet level, apparent digestibility coefficients of EPA, total n-3 PUFA, and total n-3 LC-PUFA exceeded 95% across all treatments, indicating efficient utilization irrespective of the processing method. At the ingredient level, however, the raw co-product produced the highest digestibility of EPA and total n-3 PUFA, whereas the extruded co-product produced intermediate values that were not significantly different from the raw treatment. This contrasts with Sarker et al. [22], who reported higher digestibility of EPA and other n-3 fatty acids following extrusion of *Nannochloropsis oculata* co-product, and with Le Boucher et al. [37], who observed improved PUFA digestibility under milder extrusion conditions. These contrasting responses likely reflect differences in *Nannochloropsis* species, residual lipid distribution following oil extraction, and extrusion conditions. Nevertheless, these findings indicate that extrusion at 90 °C had little influence on the utilization of residual marine omega-3 fatty acids in the QH25 co-product. Some apparent digestibility coefficients (ADCs) for fatty acid fractions in the raw ingredient exceeded 100%, including total n-3 PUFA (109.98 ± 6.66%) and EPA (101.45 ± 2.26%). Such values are unlikely to represent true biological digestibility exceeding 100% and instead likely reflect methodological limitations associated with the standard difference method used to estimate ingredient ADCs. Because ingredient ADCs are calculated indirectly from differences between the reference and test diets, relatively small analytical or experimental variations in diet-level measurements can be amplified during the calculation, particularly when the contribution of a nutrient from the test ingredient is relatively small. In addition, apparent digestibility measurements do not distinguish endogenous nutrient losses, including intestinal cellular material and digestive secretions, from undigested dietary nutrients, which may further contribute to deviations from the assumptions of the difference method [28,38]. Similar fatty acid ADCs approaching or exceeding 100% have been reported in previous aquaculture studies [26,39]. Nevertheless, we recognize that ADCs exceeding 100% represent a limitation of the calculated ingredient digestibility estimates and should not be interpreted as biologically meaningful absorption greater than 100%. Rather, these values should be regarded as mathematical overestimations and interpreted cautiously when comparing fatty acid digestibility among the tested ingredients. From a practical feed-formulation perspective, when digestible nutrient values are used for diet formulation, calculated ADCs exceeding 100% should be capped or rounded to 100%, as recommended by Glencross et al. [28], because digestibility cannot exceed complete utilization of the ingested nutrient.

Unlike extrusion, enzyme treatment did not improve nutrient digestibility and generally resulted in lower digestibility of crude protein, essential amino acids, and several fatty acid fractions. Several factors could have contributed to this response. First, the resistant cell wall of *Nannochloropsis* may have restricted enzyme access. Scholz et al. [40] characterized the cell wall of *Nannochloropsis gaditana* as a bilayer structure consisting of a cellulose-rich inner layer protected by an outer hydrophobic algaenan layer. Therefore, the protease, xylanase, and β-glucanase used in the present study may not have been sufficient to disrupt the cell wall of *Nannochloropsis* sp. QH25. However, cell-wall disruption and residual enzyme activity were not measured in the present study.

Enzyme selection and treatment conditions may also have influenced the outcome. Wu et al. [41] reported greater cell-wall degradation and lipid recovery from *Nannochloropsis* sp. when alkaline pretreatment was followed by a combination of cellulase, protease, lysozyme, and pectinase than when enzymatic treatment was applied alone. That study optimized pretreatment and enzymatic conditions, including pH, temperature, enzyme dosage, and incubation time. Although Wu et al. [41] evaluated cell-wall degradation and lipid recovery rather than nutrient digestibility, their findings suggest that pretreatment, enzyme selection, and treatment conditions can affect enzymatic disruption of *Nannochloropsis* cells. Finally, processing of the experimental diets at 90 °C exposed the enzyme-treated material to heat and pressure, which could have affected residual enzyme activity; however, enzyme activity after processing was not measured.

Sarker et al. [22], based on information provided by the enzyme manufacturer, reported that these enzymes were expected to remain stable at approximately 82–93 °C for 30 s during feed conditioning. However, Pope et al. [42] demonstrated that pelleting conditions can reduce xylanase activity, indicating that feed processing may adversely affect enzyme activity despite the expected thermal stability reported by manufacturers. In the present study, the enzyme mixture containing protease, xylanase, and β-glucanase was applied before extrusion at approximately 90 °C, and the treatment did not improve nutrient or fatty acid digestibility under the processing conditions evaluated. Because residual enzyme activity was not measured in the finished diets, it is not possible to determine whether reduced enzyme activity during extrusion contributed to the lack of a digestibility response or whether the treatment itself had limited efficacy. Therefore, the observed lack of improvement should be interpreted specifically as the outcome of pre-extrusion enzyme application under the tested processing conditions, rather than as evidence that the enzyme mixture is inherently ineffective. From a practical feed-formulation perspective, these results indicate that pre-extrusion application of the enzyme mixture did not provide a measurable digestibility advantage under the conditions evaluated. Overall, the lack of response may reflect a combination of factors, including incomplete disruption of the ingredient cell-wall matrix, the specific enzymatic treatment conditions, and possible loss of enzyme activity during extrusion.

## 5. Conclusions

Overall, the nutrient composition (Table 4 and Table 5) and digestibility (Table 6 and Table 7) results consistently demonstrate that the raw and extrusion-treated (90 °C) QH25 co-products provided comparable nutritional value for rainbow trout.

Extrusion at 90 °C resulted in only small, non-significant increases in nutrient digestibility compared with the raw co-product, whereas extrusion at 127 °C reduced crude protein, gross energy, and methionine concentrations. Precooking altered the amino acid composition of the co-product, while enzymatic processing reduced the digestibility of crude protein and amino acids under the conditions evaluated. The n-3 fatty acids remained highly digestible across the experimental diets.

Collectively, these findings indicate that the raw QH25 co-product exhibited high nutrient digestibility and that moderate extrusion offered limited additional nutritional benefit when enhancing nutrient digestibility was the primary objective. The reductions in selected nutrient concentrations at the higher extrusion temperature underscore the importance of controlling processing intensity to preserve the nutritional value of the co-product. From an aquafeed formulation perspective, the comparable digestibility of the raw and moderately extruded QH25 co-products indicates that additional extrusion processing of the ingredient may not be necessary to achieve high nutrient utilization in rainbow trout. Thus, the raw defatted QH25 co-product represents a nutritionally valuable aquafeed ingredient, and these findings provide a scientific basis for selecting processing strategies that preserve nutrient quality while avoiding unnecessary processing.

## Figures and Tables

**Table 1:** Ingredient composition of the reference diet and *Nannochloropsis* sp. QH25 co-product test diets for the digestibility experiment.

Ingredient (g/kg)	Diet			
	Ref.	70% Ref + 30% Co-Product ^a^	70% Ref + 30% Extruded Co-Product ^a^	70% Ref + 30% Enzyme-Treated Co-Product ^a^
Fish meal	300	210	210	210
Corn gluten meal	170	119	119	119
Wheat meal	174	121.8	121.8	121.8
Soybean meal	129	90.3	90.3	90.3
Wheat gluten	100	70	70	70
Vitamin/mineral premix	5	3.5	3.5	3.5
Fish oil	112	78.4	78.4	78.4
Yttrium oxide	10	7	7	7
*Nannochloropsis* sp. QH25 raw co-product	0	0	0	300
*Nannochloropsis* sp. QH25 extruded co-product	0	300	0	0
*Nannochloropsis* sp. QH25 enzyme-treated co-product	0	0	300	0

^a^ Formulated by replacing 30% of the reference diet with the respective test ingredient, resulting in a 70:30 ratio of reference diet to test ingredient.

**Table 2:** Proximate, energy, amino acid and fatty acid profiles of the reference and three test diets (enzyme, raw and extrusion processed at 90 °C) for the digestibility experiment.

Nutrient	Diet			
	Ref. ^1^	70% Ref + 30% Co-Product	70% Ref + 30% Extruded Co-Product	70% Ref + 30% Enzyme-Treated Co-Product
Proximate composition (%)				
Moisture	12.26 ± 0.06	17.14 ± 0.12	8.04 ± 0.04	15.73 ± 0.37
Protein	41.70 ± 0.08	39.95 ± 0.14	40.72 ± 0.12	40.11 ± 0.08
Fat	13.57 ± 0.08	9.06 ± 0.05	11.78 ± 0.05	10.21 ± 0.15
Fiber	1.34 ± 0.11	1.24 ± 0.21	1.42 ± 0.1	1.15 ± 0.03
Ash	9.26 ± 0.09	14.5 ± 0.13	16.83 ± 0.18	13.67 ± 0.17
Carbohydrates	23.21 ± 0.12	19.35 ± 0.16	22.63 ± 0.33	20.27 ± 0.27
Calories	3378.33 ± 1.2	2802.67 ± 11.46	3168.67 ± 4.63	2941.33 ± 5.78
Essential amino acids				
Methionine	0.75 ± 0.01	0.69 ± 0.01	0.76 ± 0.01	0.75 ± 0.02
Lysine	1.57 ± 0.01	1.76 ± 0.01	2.05 ± 0.03	1.75 ± 0.02
Phenylalanine	1.8 ± 0.01	1.79 ± 0.02	1.81 ± 0.03	1.8 ± 0.01
Leucine	3.3 ± 0.03	3.22 ± 0.02	3.41 ± 0.06	3.24 ± 0.04
Isoleucine	1.2 ± 0.02	1.34 ± 0.02	1.44 ± 0.06	1.38 ± 0.02
Threonine	1.32 ± 0.01	1.48 ± 0.02	1.63 ± 0.04	1.52 ± 0.03
Valine	1.42 ± 0.01	1.6 ± 0.03	1.75 ± 0.06	1.65 ± 0.03
Histidine	0.73 ± 0.01	0.69 ± 0	0.73 ± 0.02	0.73 ± 0.01
Arginine	2.34 ± 0.01	2.31 ± 0.04	2.49 ± 0.04	2.28 ± 0.02
Tryptophan	0.21 ± 0.01	0.32 ± 0.02	0.41 ± 0.05	0.35 ± 0.04
Fatty acid fractions (mg/g of total fatty acids)				
Total Saturated	28.49 ± 0.12	24.04 ± 0.13	29.67 ± 0.02	22.04 ± 0.14
Total Monounsat.	30.99 ± 0.16	43.2 ± 0.35	31.97 ± 0.02	47.44 ± 0.33
Total Polyunsat.	40.52 ± 0.28	32.75 ± 0.44	38.36 ± 0.02	30.52 ± 0.47
Total ω3 LCPUFA ^2^	21.76 ± 0.15	15.72 ± 0.26	19.15 ± 0.03	14.56 ± 0.27
Total ω6 LCPUFA	1.59 ± 0.01	1.43 ± 0.08	2.09 ± 0.02	1.18 ± 0.03
ω3/ω6 PUFA ^3^	1.87 ± 0.02	1.35 ± 0.02	1.64 ± 0	1.34 ± 0.02
ω3/ω6 LCPUFA	13.67 ± 0.15	11.07 ± 0.42	9.16 ± 0.11	12.37 ± 0.18
20:4ω6/20:5ω3	0.07 ± 0	0.09 ± 0	0.11 ± 0	0.08 ± 0

^1^ “Ref” indicates the complete reference diet. ^2^ LCPUFA, long chain polyunsaturated fatty acids. ^3^ PUFA, polyunsaturated fatty acids.

**Table 3:** Proximate, energy, amino acid, and fatty acid profiles of enzyme-treated, raw, and extrusion-treated *Nannochloropsis* sp. QH25 co-products used in the digestibility experiment.

Nutrient	Ingredients		
	Enzyme-Treated	Raw QH25 Co-Product	Extrusion-Treated
Proximate composition			
Moisture	6.09 ± 0.09	7.66 ± 0.07	7.11 ± 0.01
Protein	45.83 ± 0.08	45.17 ± 0.03	45.13 ± 0.11
Fat	1.19 ± 0.04	1.07 ± 0	1.41 ± 0.04
Fiber	1.74 ± 0.27	1.2 ± 0.01	0.98 ± 0.11
Ash	31.71 ± 0.04	31.11 ± 0.02	31.51 ± 0.02
Carbohydrates	15.17 ± 0.03	14.99 ± 0.02	14.84 ± 0.12
Calories	2175.33 ± 10.53	2154.67 ± 1.33	2184.67 ± 1.86
Trypsin	350 ± 11.55	866.67 ± 3.33	650 ± 40.41
Essential amino acids (% of ingredient by weight)			
Methionine	0.78 ± 0.01	1 ± 0.01	0.84 ± 0.06
Lysine	2.46 ± 0.05	2.54 ± 0.04	2.45 ± 0.02
Phenylalanine	2.06 ± 0.04	2.11 ± 0.04	2.03 ± 0.01
Leucine	3.7 ± 0.06	3.82 ± 0.06	3.68 ± 0.02
Isoleucine	1.64 ± 0.1	1.68 ± 0.06	1.58 ± 0.07
Threonine	2.12 ± 0.04	2.2 ± 0.02	2.12 ± 0
Valine	2.11 ± 0.12	2.18 ± 0.08	2.07 ± 0.09
Histidine	0.7 ± 0.03	0.7 ± 0.04	0.67 ± 0.03
Arginine	2.13 ± 0.11	2.63 ± 0.09	2.54 ± 0.05
Tryptophan	0.7 ± 0.04	0.67 ± 0.07	0.72 ± 0.01
Fatty acid fractions (% total fatty acid)			
Total Saturated	24.96 ± 0.11	22.03 ± 0.14	22.42 ± 0.07
Total Monounsaturated	46.24 ± 0.14	48.3 ± 0.14	47.99 ± 0.17
Total Polyunsaturated	28.8 ± 0.1	29.67 ± 0.26	29.59 ± 0.14
Total ω3 LCPUFA ^1^	19.83 ± 0.06	20.54 ± 0.08	20.87 ± 0.14
Total ω6 LCPUFA	5.80 ± 0.04	5.99 ± 0.12	5.68 ± 0.02
ω3/ω6 PUFA ^2^	2.24 ± 0	2.27 ± 0.03	2.40 ± 0.01
ω3/ω6 LCPUFA	3.42 ± 0.02	3.43 ± 0.05	3.67 ± 0.01

^1^ LCPUFA, long chain polyunsaturated fatty acids; ^2^ PUFA, polyunsaturated fatty acids.

**Table 4:** Effect of extrusion processing and precooking on macronutrients of *Nannochloropsis* sp. QH25 co-product (%, mean ± standard error). Values are mean and standard error of three replicate samples of treated co-product. Mean values not sharing a superscript letter in the same column differ significantly (*p* < 0.05) from Tukey’s HSD test.

	Dry Matter	Protein	Fat	Carbohydrate	Fiber	Ash	Energy
Extrusion							
**No**	93.9 ± 0.1 ^a^	45.9 ± 0.1 ^a^	1.2 ± 0.01 ^ab^	15.1 ± 0.0 ^a^	1.3 ± 0.1	31.8 ± 0.1 ^a^	2188.5 ± 4.8 ^a^
**90**	92.8 ± 0.1 ^b^	45.4 ± 0.1 ^b^	1.3 ± 0.0 ^a^	14.7 ± 0.0 ^b^	1.1 ± 0.1	31.4 ± 0.1 ^b^	2172 ± 4.8 ^a^
**127**	91.8 ± 0.1 ^c^	44.7 ± 0.1 ^c^	1.2 ± 0.0 ^ab^	14.5 ± 0.0 ^b^	0.8 ± 0.1	31.1 ± 0.1 ^b^	2148.6 ± 4.8 ^b^
Precooking							
**No**	93.0 ± 0.1	45.5 ± 0.1	1.3 ±0.0 ^a^	14.6 ± 0.0 ^b^	0.8 ± 0.1 ^b^	31.6 ± 0.1 ^a^	2185.0 ± 3.9 ^a^
**Yes**	92.7 ± 0.1	45.2 ± 0.1	1.1 ± 0.0 ^b^	14.9 ± 0.0 ^a^	1.3 ± 0.1 ^a^	31.3 ± 0.1 ^a^	2154.8 ± 3.9 ^b^
*p*-Value							
**Extrusion**	<0.001	<0.001	0.07	<0.001	0.07	<0.001	<0.001
**Precooking**	0.09	0.13	<0.001	<0.001	<0.001	0.001	<0.001
Extrusion * Precooking	0.23	0.29	0.001	0.17	0.22	0.10	0.77

* Interaction of extrusion and precooking.

**Table 5:** Effect of extrusion processing and precooking on essential amino acids of *Nannochloropsis* sp. QH25 co-product (%, mean ± standard error). Values are mean and standard error of three replicate samples of treated co-product. Mean values not sharing a superscript letter in the same column differ significantly (*p* < 0.05) from Tukey’s HSD test.

	Methionine	Lysine	Phenylalanine	Leucine	Isoleucine	Threonine	Valine	Histidine	Arginine	Tryptophan
Extrusion										
**No**	0.90 ± 0.02 ^a^	2.4 ± 0.0	2.0 ± 0.0	3.7 ± 0.0	1.5 ± 0.0	2.1 ± 0.0	2.0 ± 0.0	0.7 ± 0.00	2.5 ± 0.0	0.7 ± 0.0
**90**	0.82 ± 0.02 ^a^	2.5 ± 0.0	2.1 ± 0.0	3.8 ± 0.0	1.7 ± 0.0	2.2 ± 0.0	2.0 ± 0.0	0.7 ± 0.0	2.6 ± 0.0	0.7 ± 0.0
**127**	0.74 ± 0.02 ^b^	2.5 ± 0.0	2.1 ± 0.0	3.7 ± 0.0	1.5 ± 0.0	2.2 ± 0.0	2.2 ± 0.0	0.7 ± 0.0	2.6 ± 0.0	0.7 ± 0.0
Precooking										
**No**	0.8 ± 0.0 ^b^	2.5 ± 0.0 ^a^	2.1 ± 0.0 ^a^	3.8 ± 0.0 ^a^	1.7 ± 0.0	2.2 ± 0.0 ^a^	2.1 ± 0.0	0.7 ± 0.0 ^a^	2.6 ± 0.0 ^a^	0.7 ± 0.0
**Yes**	0.86 ± 0.0 ^a^	2.4 ± 0.0 ^b^	2.0 ± 0.0 ^b^	3.6 ± 0.0 ^b^	1.5 ± 0.0	2.1 ± 0.0 ^b^	2.0 ± 0.0	0.6 ± 0.0 ^b^	2.4 ± 0.0 ^b^	0.7 ± 0.0
*p*-Value										
Extrusion	0.001	0.46	0.39	0.64	0.09	0.21	0.09	0.11	0.08	0.91
Precooking	0.002	0.005	0.006	0.003	0.06	0.003	0.09	0.02	0.009	0.6
Extrusion * Precooking	0.003	<0.001	<0.001	0.002	<0.001	<0.001	<0.001	<0.001	0.003	0.38

* Interaction of extrusion and precooking.

**Table 6:** Apparent digestibility coefficients (%, mean ± standard error, *n* = 4) of nutrients in test ingredients (*Nannochloropsis* sp. QH25 co-product) for rainbow trout.

Nutrient	*Nannochloropsis* sp. QH25 Ingredients ^#^				
	Enzyme	Raw	Extrusion	f-Value	*p*-Value
Proximate nutrients					
Protein	79.1 ± 1.7 ^b^	83.5 ± 0.8 ^ab^	84.6 ± 0.8 ^a^	6.4	0.019
Ash	23.1 ± 5.5 ^ab^	35.3 ± 2.7 ^a^	17.9 ± 3.1 ^b^	5.1	0.033
Carbs	35.3 ± 7.8	70.6 ± 6.3	58.7 ± 6.2	3.6	0.073
Calories	76.6 ± 0.9	81.7 ± 1.5	80.4 ± 0.5	4.0	0.056
Essential Amino Acids					
Methionine	78.1 ± 1.9 ^b^	87.1 ± 1.1 ^a^	87 ± 0.7 ^a^	15.1	0.001
Lysine	73.4 ± 2.0 ^b^	86.2 ± 1.8 ^a^	83.5 ± 0.5 ^a^	18.3	<0.001
Phenylalanine	67.4 ± 2.2 ^b^	75.4 ± 3.1 ^ab^	81.2 ± 1.6 ^a^	8.4	0.009
Leucine	67.9 ± 2.6 ^b^	77.9 ± 2.7 ^a^	80.3 ± 1.3 ^a^	8.5	0.008
Isoleucine	65.7 ± 3.1 ^b^	77.1 ± 1.7 ^a^	77.0 ± 2.7 ^a^	6.3	0.019
Threonine	66.0 ± 3.4 ^b^	77.7 ± 2.1 ^a^	78.4 ± 0.7 ^a^	9.0	0.007
Valine	70.4 ± 2.1 ^b^	79.4 ± 2.5 ^ab^	84.1 ± 2.4 ^a^	9.0	0.007
Histidine	65.5 ± 3.3 ^b^	78.5 ± 3.8 ^a^	82.2 ± 1.5 ^a^	8.6	0.008
Arginine	73.6 ± 2.7 ^b^	86.1 ± 3.3 ^a^	87.5 ± 1.7 ^a^	8.5	0.008
Tryptophan	60.9 ± 3.6 ^b^	81.0 ± 1.0 ^a^	76.1 ± 2.6 ^a^	15.8	0.001
Cystine	64.1 ± 5.1	65.4 ± 2.2	51.9 ± 2.3	4.6	0.054
Fatty acid fractions (% of total fatty acids) *					
Total n3 PUFA	65.8 ± 17.1 ^b^	110.0 ± 6.7 ^a^	75.4 ± 5.2 ^ab^	4.4	0.046
EPA	78.3 ± 7.6 ^b^	101.5 ± 2.3 ^a^	88.5 ± 3.0 ^ab^	5.6	0.027
Total n3 LCPUFA	67.0 ± 15.4	107.1 ± 5.9	82.2 ± 5.8	4.0	0.057

^#^ Mean values sharing at least one common letter within the same row are not significantly different (*p* > 0.05), whereas mean values with no common letter differ significantly (Tukey’s HSD, *p* < 0.05). * n3 PUFA, omega 3 polyunsaturated fatty acids; EPA, eicosapentaenoic acid; n3 LCPUFA, omega 3 long chain polyunsaturated fatty acids.

**Table 7:** Apparent digestibility coefficients (%, mean ± standard error, *n* = 4) of nutrients in feed (reference, enzyme, raw, and extruded) for rainbow trout.

Nutrient	Diet Digestibility ^#^					
	Reference	Enzyme	Raw	Extrusion	f-Value	*p*-Value
Proximate composition						
Protein	93.16 ± 0.24 ^a^	88.49 ± 0.39 ^c^	89.92 ± 0.36 ^b^	90.32 ± 0.22 ^b^	39.766	<0.001
Ash	33.45 ± 3.37 ^ab^	25.36 ± 3.72 ^ab^	35.02 ± 2.52 ^a^	21.04 ± 2.21 ^b^	4.851	0.02
Carbs	53.14 ± 2.45 ^ab^	50.51 ± 2.15 ^b^	60.07 ± 1.85 ^a^	57.03 ± 1.58 ^ab^	4.302	0.028
Calories	83.27 ± 0.98 ^a^	78.23 ± 1.14 ^b^	81.87 ± 1.09 ^ab^	80.89 ± 0.36 ^ab^	5.056	0.017
Essential amino acids						
Methionine	93.76 ± 0.06 ^a^	88.23 ± 0.23 ^b^	91.49 ± 0.22 ^ab^	91.44 ± 0.11 ^ab^	26.227	<0.001
Lysine	82.86 ± 0.13 ^a^	77.9 ± 0.23 ^b^	83.95 ± 0.45 ^a^	82.93 ± 0.03 ^a^	19.613	<0.001
Phenylalanine	88.58 ± 0.16 ^a^	80.27 ± 0.17 ^b^	83.48 ± 0.54 ^ab^	85.44 ± 0 ^ab^	27.261	<0.001
Leucine	89.63 ± 0.2 ^a^	81.14 ± 0.14 ^b^	85.2 ± 0.43 ^ab^	86.07 ± 0.05 ^ab^	29.029	<0.001
Isoleucine	84.54 ± 0.07 ^a^	75.67 ± 0.32 ^b^	80.98 ± 0.3 ^a^	80.43 ± 0.06 ^a^	18.405	<0.001
Threonine	85.3 ± 0.19 ^a^	75.71 ± 0.59 ^b^	81.65 ± 0.66 ^a^	81.94 ± 0.05 ^a^	16.471	<0.001
Valine	80.72 ± 1.07 ^ab^	75.67 ± 0.17 ^b^	79.42 ± 0.75 ^ab^	82.17 ± 0.1 ^a^	4.657	0.022
Histidine	87.22 ± 0.2 ^a^	79.28 ± 0.35 ^b^	83.8 ± 0.48 ^a^	85.21 ± 0.17 ^a^	16.593	<0.001
Arginine	86.38 ± 0.41 ^a^	82.4 ± 0.21 ^b^	86.29 ± 0.64 ^a^	87.25 ± 0.04 ^a^	7.222	0.005
Tryptophan	78.05 ± 0.2 ^a^	67.92 ± 1.24 ^b^	80.06 ± 0.38 ^a^	75.17 ± 0.35 ^a^	9.532	0.002
Cystine	90.74 ± 0.15 ^a^	82.6 ± 0.6 ^b^	82.59 ± 0.35 ^bc^	79.3 ± 0.39 ^c^	30.918	<0.001
Fatty acid fractions (% of total fatty acids) *						
Total n3 PUFA	98 ± 0.2 ^ab^	96 ± 0.1 ^b^	98.4 ± 0.1 ^a^	97 ± 0.2 ^b^	6.54	0.007
EPA	98.6 ± 0.1 ^a^	96.5 ± 0.1 ^b^	98.8 ± 0.1 ^a^	97.7 ± 0.1 ^ab^	9.89	0.001
Total n3 LCPUFA	98 ± 0.2 ^a^	95.9 ± 0.1 ^b^	98.3 ± 0.1 ^a^	97.1 ± 0.1 ^ab^	6.945	0.006

^#^ Mean values sharing at least one common letter within the same row are not significantly different (*p* > 0.05), whereas mean values with no common letter differ significantly (Tukey’s HSD, *p* < 0.05). * n3 PUFA, omega 3 polyunsaturated fatty acids; EPA, eicosapentaenoic acid; n3 LCPUFA, omega 3 long chain polyunsaturated fatty acids.

## Data Availability

All data generated for this study have been submitted at https://doi.org/10.15482/USDA.ADC/32794959.
